# Adjuvant Chemotherapy in the Treatment of Intraductal Papillary Mucinous Neoplasms of the Pancreas: Systematic Review and Meta-Analysis

**DOI:** 10.1007/s00268-021-06309-8

**Published:** 2021-09-20

**Authors:** Eric Chong, Bathiya Ratnayake, Bobby V. M. Dasari, Benjamin P. T. Loveday, Ajith K. Siriwardena, Sanjay Pandanaboyana

**Affiliations:** 1 Faculty of Medical and Health Sciences, Surgical and Translational Research Centre, University of Auckland, Auckland, New Zealand; 2 Hepatobiliary and Transplant Unit, Queen Elizabeth Hospital, Birmingham, UK; 3 Hepatobiliary and Upper Gastrointestinal Unit, Royal Melbourne Hospital, Victoria, Australia; 4 Department of Surgical Oncology, Peter MacCallum Cancer Centre, Victoria, Australia; 5 Hepatobiliary and Pancreatic Unit, Manchester Royal Infirmary, Manchester, UK; 6 Pancreatic and Transplant Surgery, HPB and Transplant Unit, Department of Hepatobiliary, Freeman Hospital, Newcastle upon Tyne, UK; 7 Population Health Sciences, Newcastle University, Newcastle upon Tyne, UK

## Abstract

**Background:**

The present systematic review aimed to compare survival outcomes of invasive intraductal papillary mucinous neoplasms (IIPMNs) treated with adjuvant chemotherapy versus surgery alone and to identify pathologic features that may predict survival benefit from adjuvant chemotherapy.

**Method:**

A systematic search of MEDLINE, PubMed, Scopus, and EMBASE was performed using the PRISMA framework. Studies comparing adjuvant chemotherapy and surgery alone for patients with IIPMNs were included. Primary endpoint was overall survival (OS). A narrative synthesis was performed to identify pathologic features that predicted survival benefits from adjuvant chemotherapy.

**Results:**

Eleven studies and 3393 patients with IIPMNs were included in the meta-analysis. Adjuvant chemotherapy significantly reduced the risk of death in the overall cohort (HR 0.57, 95% CI 0.38–0.87, *p* = 0.009) and node-positive patients (HR 0.29, 95% CI 0.13–0.64, *p* = 0.002). Weighted median survival difference between adjuvant chemotherapy and surgery alone in node-positive patients was 11.6 months (95% CI 3.83–19.38, *p* = 0.003) favouring chemotherapy. Adjuvant chemotherapy had no impact on OS in node-negative patients (HR 0.53, 95% CI 0.20–1.43, *p* = 0.209). High heterogeneity (*I*^2^ > 75%) was observed in pooled estimates of hazard ratios. Improved OS following adjuvant chemotherapy was reported for patients with stage III/IV disease, tumour size > 2 cm, node-positive status, grade 3 tumour differentiation, positive margin status, tubular carcinoma subtype, and presence of perineural or lymphovascular invasion.

**Conclusion:**

Adjuvant chemotherapy was associated with improved OS in node-positive IIPMNs. However, the findings were limited by marked heterogeneity. Future large multicentre prospective studies are needed to confirm these findings and explore additional predictors of improved OS to guide patient selection for adjuvant chemotherapy.

**Supplementary Information:**

The online version contains supplementary material available at 10.1007/s00268-021-06309-8.

## Introduction

Intraductal papillary mucinous neoplasms (IPMNs) are mucin-producing epithelial neoplasms of the pancreas originating from the main pancreatic duct and/or one or more of its tributaries. The worldwide prevalence of incidentally detected IPMNs is rising secondary to the widespread utilisation of cross-sectional abdominal imaging [1]. Historical concerns regarding the likely overestimated malignant potential of IPMNs meant indiscriminate resection. However, through experience and an improved understanding of the pathophysiology, IPMNs are now understood to be a spectrum of disease whereby low-risk selected patients perform well when a conservative surveillance approach is employed [2]. Indeed, IPMNs are distributed into low-grade dysplasia, intermediate-grade dysplasia, high-grade dysplasia, and invasive carcinoma [3]. High-risk clinical and radiological stigmata considered as predictors of high-grade dysplasia or invasive carcinoma include obstructive jaundice, enhancing mural nodule ≥5 mm, and main pancreatic duct (MPD) ≥ 10 mm, while worrisome features include cyst ≥3 cm, enhancing mural nodule <5 mm, MPD 5–9 mm, abrupt change in MPD diameter with distal pancreas atrophy, lymphadenopathy, elevated CA 19–9, and cyst growth of > 5 mm/2 year [2, 4].

Patients deemed to be at high risk would undergo pancreatic resection; however, the role of adjuvant chemotherapy is not standardised in patients with invasive IPMNs (IIPMNs) on post-operative histology. While the European Study Group on Pancreatic Cystic Neoplasms recommended adjuvant chemotherapy for IIPMNs with or without lymph node involvement [5], the revised Fukuoka consensus guidelines made no recommendations on adjuvant chemotherapy [2]. Currently, there remain no quantitative data to guide the use of adjuvant chemotherapy and prior systematic review is limited to narrative synthesis of historic literature [6]. The current systematic review and meta-analysis aimed to review the survival outcome of adjuvant chemotherapy compared to surgery alone for the treatment of patients with IIPMNs who underwent pancreatic resection and to identify pathologic features that may predict survival benefit from adjuvant chemotherapy.

## Method

### Study selection

The study was performed according to the Preferred Reporting for Systematic Reviews and Meta-analysis (PRISMA) guidelines [7]. A systematic search was performed on 5 February 2021 using four databases: PubMed, MEDLINE, Embase, and Scopus. A detailed analysis of the search strategy including the database specific syntax is reported in the Appendix. Reference lists of studies included in the full-text review were reviewed to identify additional articles not captured in the original search strategy.

### Eligibility criteria

Two authors (EC and BR) independently screened the title and abstract of studies to identify relevant studies. Articles were included if they compared pancreatic resection followed by adjuvant chemotherapy versus pancreatic resection alone for IIPMNs in adults. Exclusion criteria were case reports, editorials, review articles, and non-English articles, and studies with less than five participants were excluded. Studies including IPMNs with concomitant pancreatic ductal adenocarcinoma (PDAC) were also excluded. Any enduring disagreement in study selection was adjudicated by the senior author (SP).

### Critical appraisal

Two authors (EC and BR) independently performed the quality assessment using the ROBINS-1 tool [8]. Each study was assessed in seven different domains for biases that could occur in non-randomised studies. The domains were categorised as pre-intervention, during intervention, or post-intervention and graded as low, moderate, high, or critical risk of bias. An overall risk of bias was decided based on the assessments of risk of bias in individual domains [8]. Differences in quality assessment were discussed between the two authors (EC and BR). Again, enduring differences in quality assessments were adjudicated by senior author (SP).

### Data extraction

Two authors (EC and BR) independently performed the data extraction for this study. Data extracted included study characteristics (study design, country, number of patients, follow-up duration) and patient characteristics (age, type of adjuvant chemotherapy and radiotherapy, cancer stage, tumour size, nodal status, tumour grading, margin status, invasive carcinoma subtype, perineural and lymphovascular invasion). Data on survival outcomes of the adjuvant treatment and surgery alone groups were also extracted.

### Terminology and definitions

Adjuvant chemotherapy referred to chemotherapy administered in adjuvant setting with or without additional radiotherapy.

Surgery alone referred to patients who did not receive adjuvant chemotherapy.

Invasive carcinoma subtypes referred to histology of invasive component of IPMNs and included tubular carcinoma, colloid carcinoma, and oncocytic carcinoma [3]. Precursor epithelial type referred to histology of preinvasive IPMNs and included pancreatobiliary type, gastric type, intestinal type, and oncocytic type [3].

TNM staging was defined according to the Union for International Cancer Control 6th edition [9] or American Joint Committee on Cancer 5th–8th editions for pancreatic cancer [10–15].

Node positivity was determined on histological examination and was defined as one or more nodes with lymph node involvement via direct extension or metastasis.

Positive resection margin included macroscopic (*R*2) or microscopic invasion of the margin, or a tumour-free margin of < 1 mm (*R*1).

Overall survival (OS) was defined as the duration between the date of diagnosis [13] or surgery [10, 14–16] and death or loss to follow-up.

The primary outcome measure was pooled hazard ratios for OS and weighted median survival difference between adjuvant chemotherapy and surgery alone. The secondary outcomes were pathologic predictors of survival benefit from adjuvant chemotherapy in IIPMNs.

### Statistical analysis

Statistical analysis was performed using RStudio with the following packages: meta, metafor, dmetar, and tidyverse (*R* Foundation for Statistical Computing, Austria 2014) [17–20]. Data derived from study employing propensity score analysis were preferentially extracted where overlapping series existed. Pooled hazard ratio for the comparison between adjuvant chemotherapy and surgery alone was performed only when three or more sets of data are available using multivariate analysis data where reported. Weighted median survival difference between adjuvant chemotherapy and surgery alone was also estimated. Standard error was estimated using confidence interval and *p* value [21]. Studies were weighted using the generic inverse variance method [22], and tau^2^ was estimated using the Sidik–Jonkman method [23]. A random effect model was used in the meta-analysis. Statistical heterogeneity was determined using *I*^2^ value. *I*^2^ thresholds of 25, 50, and 75% indicated low, moderate, and high heterogeneity. Heterogeneity was non-significant when *I*^2^ < 25% [24].

## Results

### Study characteristics

**Fig. 1:**
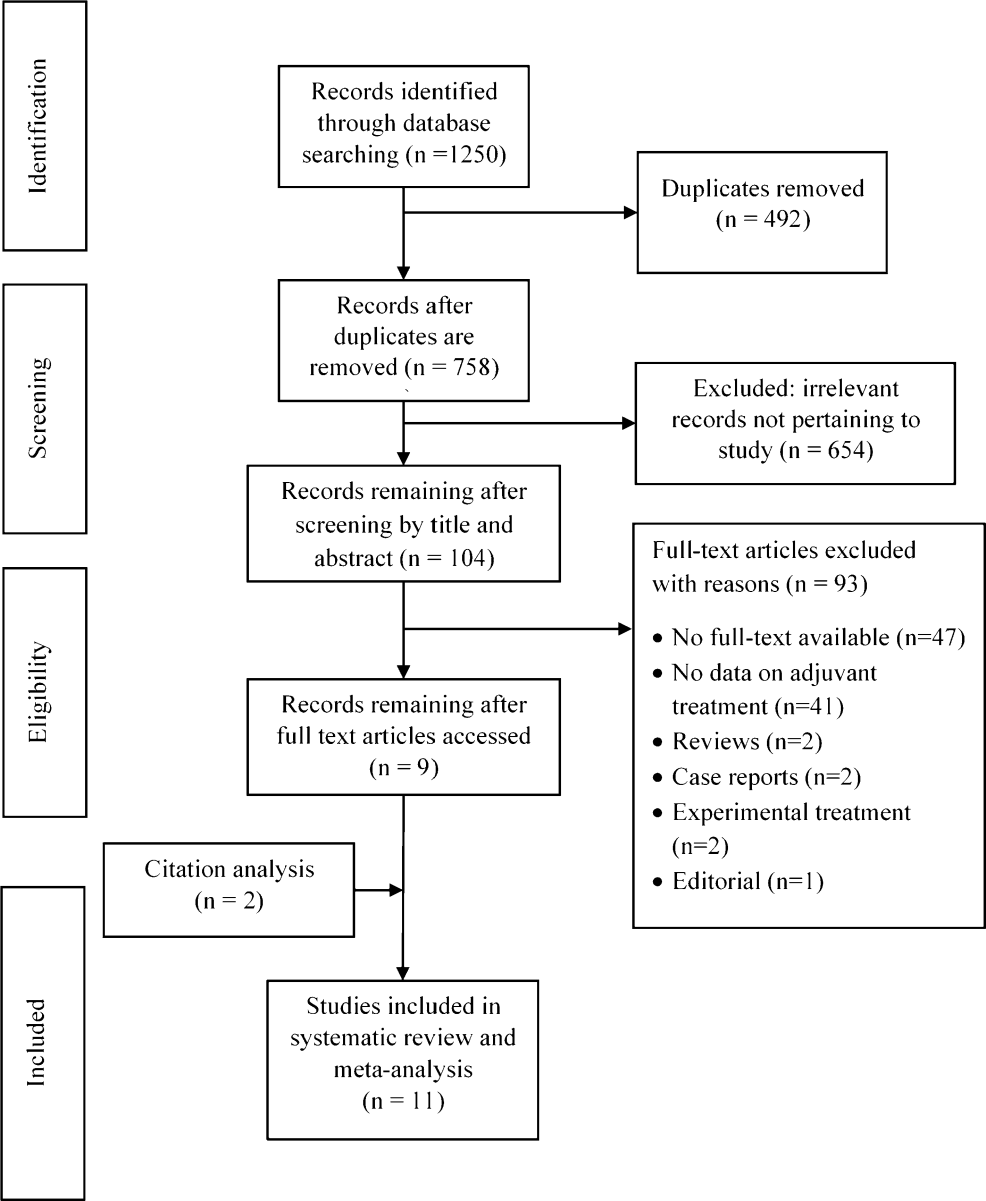
Prisma flow chart of literature search strategy

The systematic search of databases returned 1250 articles. Eleven studies met the inclusion criteria and were included in the meta-analysis (Fig. 1). The studies were published between 2008 and 2020. All studies were retrospective in nature. Studies were performed in the USA (*n* = 7) [9, 10, 13, 15, 16, 25, 26], Italy (*n* = 2) [11, 14], France (*n* = 1) [12], and Japan (*n* = 1) [27]. The total number of patients from the included studies was 3393, and all patients had the diagnosis of IIPMNs. IIPMNs diagnosis was made with clear exclusion of IPMNs with concomitant PDAC in three studies [10, 11, 27]. In the remainder of eight studies, IPMNs with concomitant PDAC were not clearly excluded when diagnosing IIPMNs [9, 12–16, 25, 26]. Overall, 1535 patients received adjuvant chemotherapy and 1858 patients received surgery alone (Table 1). The weighted median follow-up duration was 86 months (95% CI 57.3–86 months).

**Table 1:** Characteristics of studies, the number of adjuvant therapies, and type of chemotherapy

Author (year)	Study design	Country	Included patients			Surgery type	Type of AT	Type of AC	Study duration	Follow-up duration*
			AT	No AT	Total					
Hirono et al. [27]	Retrosp.	Japan	88	159	247	NR	NR	NR	1996–2014	54.2 (0.2–241.2)
Mungo et al. [25]	Retrosp.	USA	225	267	492	DP (163/492), PD (242/492), TP (79/492)	AC only (138/225), AC + RT (87/225)	NR	2006–2015	57.3 (31.4–100.6)^†^
Rodrigues et al. [10]	Retrosp.	USA	34	69	103	DP (28/103), PD (60/103), TP (13/103), other (2/103)	AC only (15/34) AC + RT (19/34)	GEM (30/34), GEM–capecitabine (2/34), 5-FU (2/34)	Jan 1993–Sept 2018	47 (6–274)
Marchegiani et al. [11]	Retrosp.	Italy	19	83	102	DP (23/102), PD (59/102), TP (20/102)	AC only (14/19) AC + RT (5/19)	GEM (15/19), GEM + OXA (2/19), 5-FU + OXA (2/19)	1990–2016	72 (5–318)
Duconseil et al. [12]	Retrosp.	France	61	22	82	NR	AC	NR	1 Jan 2006–31 Dec 2012	28^‡^
McMillan et al. [13]	Retrosp.	USA	953	1074	2027	DP (349/2027), PD (1403/2027), TP (275/2027)	AC only (293/953), AC + RT (660/953);	Single-agent AC (609/953), multiagent chemotherapy (244/953), unknown (100/953)	1998–2010	86 (69–116)
Caponi et al. [14]	Retrosp.	Italy	33	31	64	NR	AC only (23/33) AC + RT (10/33)	GEM (33/33)	2005–Jun 2011	NR
Alexander et al. [26]	Retrosp.	USA	17	27	44	NR	CRT	Infusional 5-FU (11/19), bolus 5-FU (4/19), capecitabine (1/19), 5-FU/GEM (1/19); 5 received additional AC (5-FU (3/5) and GEM (2/5))	1990–2005	19 (1–145)
Swartz et al. [16]	Retrosp.	USA	40	30	70	DP (11/70), PD (59/70)	CRT	Most received 5-FU-based AC + RT	1999–2004	24.8
Turrini et al. [15]	Retrosp.	USA	37	61	98	DP (19/98), PD (62/98), TP (17/98)	AC (7/37), AC + RT (30/37)	5-FU-based (28/37) GEM-based (9/37)	1 Jan 1989–31 Dec 2006	32 (12–180) ^‡^
Schnelldorferet al. [9]	Retrosp.	USA	28	35	63	NR	CRT	NR	1992–2005	NR

*5-FU* 5-fluorouracil*, AC* adjuvant chemotherapy, *AT* adjuvant therapy (including adjuvant chemotherapy ± radiotherapy), *CRT* chemoradiotherapy, *DP* distal pancreatectomy, *GEM* gemcitabine, *No AT* surgery alone, *NR* not reported, *OXA* oxaliplatin, *PD* pancreaticoduodenectomy, *Retrosp.* retrospective cohort study, *RT* radiotherapy, *TP* total pancreatectomy, *USA* United States of America

^*^Reported in median (range) and months, unless denoted otherwise

^†^Reported in median (IQR),

^‡^Reported in mean

### Tumour characteristics

Pre-operatively, main duct, branch duct, and mixed-type IPMNs were observed in 44.2% (144/326), 12.6% (41/326), and 42.6% (139/326) patients, respectively. Type of ductal involvement in IPMNs was unknown in 0.6% (2/326) patients. The most common surgery performed was pancreatoduodenectomy (65.2%, 1885/2891), followed by distal pancreatectomy (20.5%, 593/2891), total pancreatectomy (13.9%, 403/2891), and others (0.3%, 10/2891). Following resection, margin was positive in 19.8% (596/3018) and negative (*R*0) in 77.3% (2334/3018) patients. Margin status was unknown in 2.9% (88/3018) patients. Most tumours were stage I or II (86.0%, 2619/3046). Tumour size of 19.1% (307/1611) patients was < 2 cm, and that of 78.4% (1263/1611) patients was > 2 cm. Node-positive status was observed in 39.6% (1263/3187) of patients.

Invasive subtype was most commonly tubular carcinoma in 64.3% (331/515) patients, followed by colloid carcinoma and oncocytic carcinoma in 34.2% (176/515) and 1.6% (8/515) patients, respectively. The precursor epithelial type of IIPMNs was predominantly pancreatobiliary (56.8%) in one study. Precursor epithelial type was not reported in other studies. Tumour histology was graded as *G*1 or 2 in 70.1% (1766/2519) patients and as *G*3 or 4 in 23.5% (591/2519) patients. Tumour grade was unknown in 6.4% (162/2519) patients. Perineural and lymphovascular invasions were seen in 44.6% (205/460) and 23.6% (87/369) patients, respectively.

### Adjuvant chemotherapy

The type of adjuvant chemotherapy was reported in five studies including 140 patients [10, 11, 14, 15, 26]. Gemcitabine-based adjuvant chemotherapy was utilised for 65.0% (91/140) patients, 5-fluorouracil (5-FU)-based for 33.6% (47/140) patients, 5-FU/gemcitabine for 0.7% (1/140) patients, and capecitabine alone for 0.7% (1/140) patients. Eight studies reported the use of additional adjuvant radiotherapy in addition to chemotherapy [10, 11, 13–16, 25, 26], in 61.2% patients (868/1419) (Table 1).

Enrolled patients who received adjuvant chemotherapy tended to be younger [10, 13, 25] and presented with stage II disease and above [13, 16], larger tumour size [13, 25], node-positive status [10, 13, 15, 16, 25, 26], poorly differentiated or undifferentiated tumour [25], positive resection margin [13], and tubular carcinoma as invasive component [16] (Table 2). Weighted comparisons of adjuvant chemotherapy and surgery alone showed significantly more patients receiving adjuvant chemotherapy presented with node-positive status (55.3% vs. 27.5%, *p* < 0.0001), *G*3 tumour differentiation (22.2% vs. 17.1%, *p* = 0.001), and perineural invasion (60.4% vs. 32.5%, *p* = 0.038). No differences were observed for disease stage, tumour size, invasive carcinoma subtype, margin status, and lymphovascular invasion.

**Table 2:** Comparisons of baseline characteristics of patients with invasive IPMN

Author	Median age (AT vs. no AT)	Stage I–II/III–IV, % (AT vs. no AT)	Tumour size < 2/ > 2 cm, % (AT vs. no AT)	Lymph node involvement, % (AT vs. no AT)	Tumour grading G1-2/G3-4, % (AT vs. no AT)	Positive margin, % (AT vs. no AT)	Tubular carcinoma, % (AT vs. no AT)
Hirono et al. [27]	NR	35.3/41.7 vs 64.7/58.3*	NR	26.9/73.1 vs 55.3/44.7*	NR	NR	54.8 vs 45.2
Mungo et al. [25]	NR*	NR	35.7/51.3 vs 64.3/48.7*	44.89 vs 13.48*	57.8/20.0 vs 49.1/12.4*	15.56 vs 9.36	NR
Rodrigues et al. [10]	62 vs 74*	NR	NR	50 vs 27.5*	21.4 vs 12.5‡	14.7 vs 10.1	70.6 vs 58.0
Marchegiani et al. [11]	66 vs 66	NR	NR	62.3 vs 38.6	68.3/31.6 vs 74.6/25.4	21.1 vs 9.8	42.1 vs 50.7
Duconseil et al. [12]	NR	NR	NR	NR	NR	NR	NR
McMillan et al. [13]	NR*	74.2/25.5 vs 84.7/15.3*	34.8/47.7 vs 65.2/52.3*	63.1 vs 36.9*	57.0/16.6 vs 21.6/56.2	59.6 vs 40.4*	NR
Caponi et al. [14]	67 vs 71	NR	NR	81.8 vs 41.9	9.7 v 12.1‡	NR	93.50 vs 97.0
Alexander et al. [26]	NR	94.1/5.9 vs 100/0	NR	52.9 vs 18.5*	NR	35.3 vs 18.5	NR
Swartz et al. [16]	NR	80 vs 46.7*†	NR	65.0 vs 30*	30.0 vs 16.7‡	20.0 vs 10.0	65.0 vs 26.7*
Turrini et al. [15]	NR	NR	NR	65 vs 25*	60/42 vs 47/54	19 vs 3*	NR
Schnelldorfer et al. [9]	NR	NR	NR	NR	NR	NR	NR

*AT* adjuvant therapy (adjuvant chemotherapy ± radiotherapy), *G* grade, *NR* not reported

^*^ Statistically significant difference between group receiving adjuvant therapy and surgery alone

^†^ Reported as percentage of tumours at stage II/III

^‡^ Reported as percentage of tumour graded as grade3

### Primary outcome measure

#### Overall survival

**Fig. 2:**
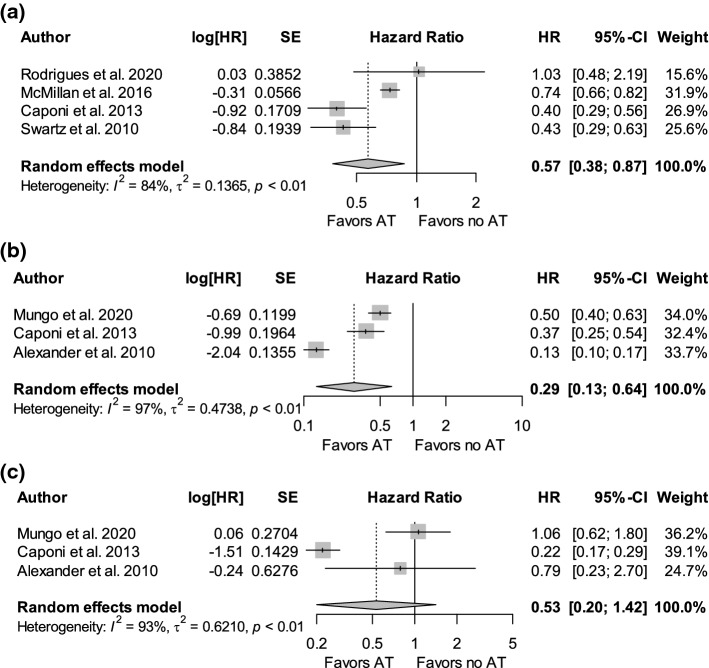
Forest plot of pooled hazard ratios of overall survival in patients with invasive intraductal papillary mucinous neoplasms treated with adjuvant chemotherapy versus surgery alone in **a** overall cohort, **b** node-positive patients, and **c** node-negative patients. AT adjuvant treatment, HR hazard ratio, log[HR] log of hazard ratio, SE standard error of treatment effect

**Fig. 3:**
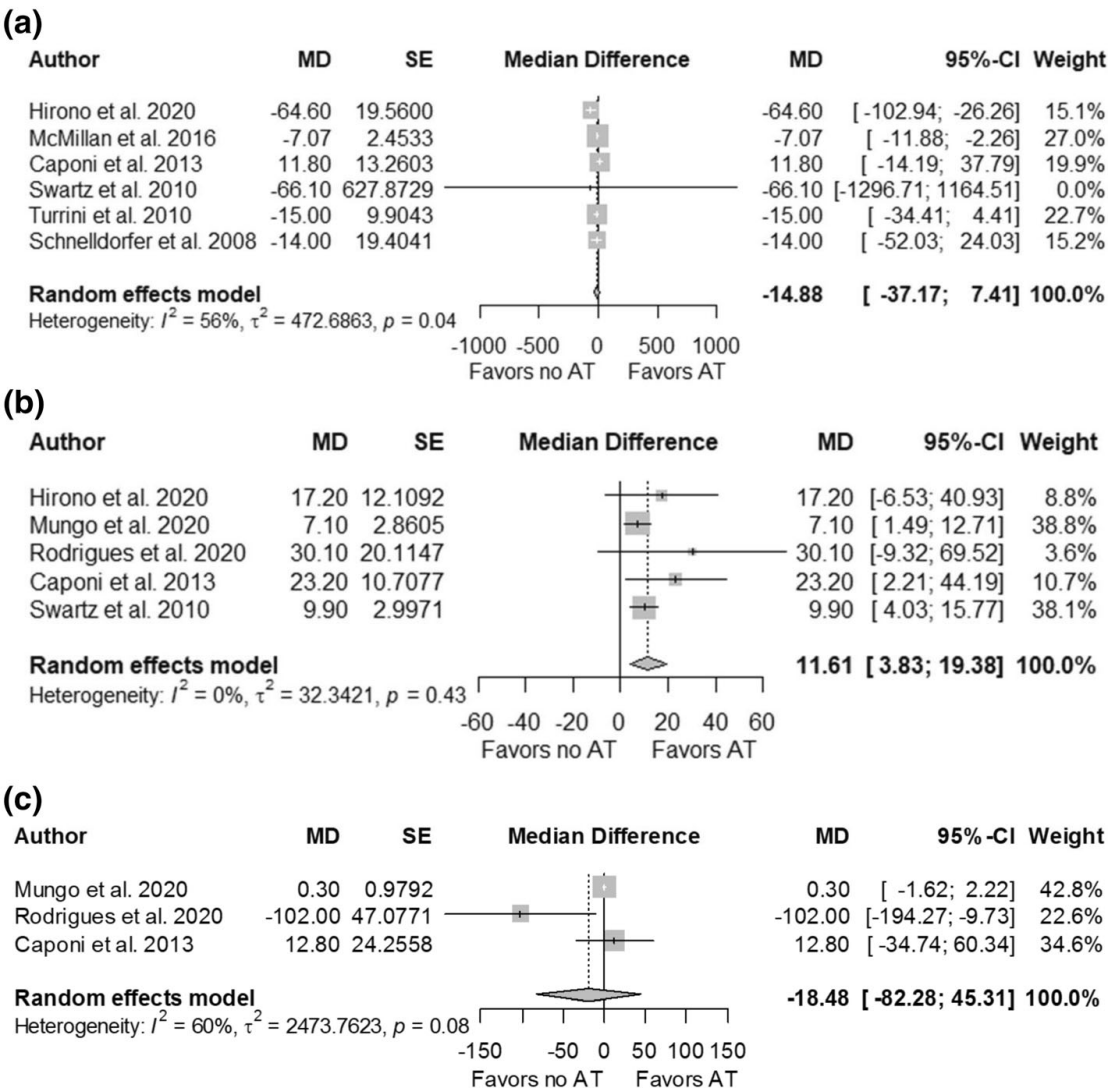
Forest plot of median difference of survival time in patients with invasive intraductal papillary mucinous neoplasms treated with adjuvant chemotherapy versus surgery alone in **a** overall cohort, **b** patients with nodal involvement, and **c** patients with no nodal involvement. AT adjuvant treatment, MD median difference, TE treatment effect, seTE standard error of treatment effect

Seven studies reported the impact of adjuvant chemotherapy on OS in 2924 patients with IIPMNs [10, 13, 14, 16, 25–27]. Adjuvant chemotherapy group included 43.7% (1277/2924) patients, while surgery alone group included 56.3% (1647/2924) patients. Adjuvant chemotherapy significantly reduced the risk of death by 43% (HR 0.57, 95% CI 0.38–0.87, *p* = 0.009) (Fig. 2a). There was no difference in weighted median survival time between adjuvant chemotherapy group and surgery alone group (-14.9 months, 95% CI − 37.17–7.41, *p* = 0.191) (Fig. 3a).

### Secondary outcome measures

#### Impact of adjuvant chemotherapy on node-positive and node-negative groups

Six studies reported the impact of adjuvant chemotherapy in 324 patients with IIPMNs with nodal involvement [10, 14, 16, 25–27]. Adjuvant chemotherapy group included 65.7% (213/324) patients, and surgery alone group included 34.3% (111/324) patients. Adjuvant chemotherapy significantly reduced the risk of death by 71% (HR 0.29, 95% CI 0.13–0.64, *p* = 0.002) (Fig. 2b). Weighted median survival difference was 11.6 months (95% CI 3.83–19.38, *p* = 0.003) between the two groups favouring patients who received adjuvant chemotherapy (Fig. 3b).

Three studies reported the impact of adjuvant chemotherapy in 409 patients with node-negative IIPMNs [14, 25, 26]. Adjuvant chemotherapy group included 33.7% (138/409) patients, and surgery alone group included 66.3% (271/409) patients. There was no difference in risk of death between adjuvant chemotherapy and surgery alone in node-negative patients (HR 0.53, 95% CI 0.20–1.43, *p* = 0.209) (Fig. 2c). Similarly, there was no difference in weighted median survival between the two groups (-18.5 months, 95% CI -82.38–45.31, *p* = 0.570) (Fig. 3c).

#### Pathologic features of invasive IPMN that predicted survival benefit from adjuvant chemotherapy

Table 3 summarises pathologic features of IIPMNs that predicted a survival benefit from adjuvant chemotherapy as reported in the literature. A meta-analysis of these pathologic features was not performed due to under-reporting or variations in survival analysis methodology resulting in inadequate data sets.

**Table 3:** Association between pathologic features and survival benefit following adjuvant chemotherapy on univariate and multivariate analysis

Author	Survival benefits favouring adjuvant chemotherapy*									
	Overall	Stage III/IV	Tumour size**	N +	N-	G3	Positive margin	Tubular carcinoma	Perineural invasion	Lymphovascular invasion
Hirono et al. [27]	No	No		No	No			No		
Mungo et al. [25]				Yes^§^	No^§^					
Rodrigues et al. [10]	No^§^			No	No^**¶**^			No		
Marchegiani et al. [11]	No^†^			Yes^†^		No^†^	No^†^	Yes^†^		
Duconseil et al. [12]	Yes				No					
McMillan et al. [13]	Yes^§^	Yes^§^	Yes^§^	Yes^§^	No	Yes^§^	Yes^§^			
Caponi et al. [14]	Yes^§^			Yes	Yes					
Alexander et al. [26]				Yes^‡^					Yes^‡^	Yes^‡^
Swartz et al. [16]	Yes^§^			Yes			Yes			
Turrini et al. [15]	Yes			No	No		No			
Schnelldorfer et al. [9]	No									

*Yes* indicated significant (*p* < 0.05) survival benefit following the use of adjuvant chemotherapy

*No* indicated survival benefit did not favour adjuvant chemotherapy

*N* + regional lymph node involvement, *N-* no lymph node involvement

*survival outcome was measured as overall survival unless indicated otherwise

† survival outcome was cancer-/disease-specific survival

‡ indicates survival outcomes included overall survival and cancer-/disease-specific survival

§ indicates multivariable or propensity-weighted analysis was used in comparison

**¶** indicates survival benefits favoured surgery alone

** Comparing tumour size < 2 cm versus >2 cm

Eight pathologic features were associated with a survival benefit following adjuvant chemotherapy on univariate analysis. Eight studies assessed the role of adjuvant chemotherapy in node-positive patients [10, 11, 14–16, 25–27], and five studies found a survival benefit with adjuvant chemotherapy [11, 14, 16, 25, 26]. Three studies assessed the role of adjuvant chemotherapy in patients with positive resection margin [11, 15, 16], and one found a survival benefit compared to those who underwent surgery alone [16]. Three studies assessed the role of adjuvant chemotherapy in patients with tubular carcinoma [10, 11, 27], and only one found survival benefit with adjuvant chemotherapy [11]. Perineural invasion and lymphovascular invasion were examined in one study, and both features were associated with survival benefit after adjuvant chemotherapy compared to surgery alone [13, 26]. *G*3 tumour differentiation was not associated with survival benefit after chemotherapy in one study [11].

Multivariate or propensity-weighted analysis was employed by two studies to distinguish treatment effect of adjuvant chemotherapy [13, 25]. OS in node-positive patients was significantly improved after adjuvant chemotherapy in both studies [25]. Survival benefit was also seen in stage III/IV disease, tumour size > 2 cm, and grade 3 tumour differentiation in one study [13].

### Quality assessment

Results of quality assessment using ROBINS-1 tool are given in Supplementary Table 1. Overall risk of bias was moderate in five studies [11, 14–16, 25], serious in four studies [10, 13, 26, 27], and critical in two studies [9, 12]. Studies tended to score poorly in bias due to confounding. The risk of bias due to confounding was critical in two studies [9, 12], serious in four other studies [10, 13, 26, 27], and moderate in five other studies [11, 14–16, 25]. Included studies generally performed well in six other biases assessed with ROBINS-I tool.

High heterogeneity (*I*^2^ > 75%) was observed in pooled estimates of hazard ratios in the overall comparison (*I*^2^ = 84%), node-negative comparison (*I*^2^ = 93%), and node-positive comparison (*I*^2^ = 97%). The sources of heterogeneity were Rodrigues et al. in the overall comparison [10], Caponi et al. in node-negative comparison [14], and Alexander et al. in node-positive comparison [26]. In contrast, heterogeneity in weighted median survival difference was insignificant in overall comparison and node-positive comparison (*I*^2^ = 0%) and moderate in node-negative comparison (*I*^2^ = 60%).

## Discussion

The present systematic review and meta-analysis included 3393 patients from 11 studies and assessed the impact of adjuvant chemotherapy in IIPMNs. Adjuvant chemotherapy after pancreatic resection was associated with improved OS in node-positive IIPMNs with a survival advantage of 11.6 months. In contrast, adjuvant chemotherapy had no effect on risk of death or weighted median survival in node-negative patients. The narrative synthesis identified eight pathologic features of IIPMNs that predicted improved survival following adjuvant chemotherapy. These include stage III/IV disease, tumour size > 2 cm, node-positive status, positive margin status, high-grade histology, tubular carcinoma subtype, and perineural or lymphovascular invasion.

A previous systematic review of eight studies found five IIPMNs features (node-positive status, stage, positive margin, histological grade, invasive carcinoma subtype) that benefited from adjuvant chemotherapy. However, these findings were based on narrative data without quantitative analysis. In the present review, an improved OS in node-positive patients undergoing adjuvant chemotherapy was observed on pairwise analysis, whereas similar OS was found in node-negative patients when compared with surgery alone. Node positivity may be an indicator of further systemic micrometastasis, a likely target of systemic therapy [28, 29]. Indeed, lymph node metastasis as well as disease stage was associated with extra-pancreatic recurrence [27, 30]. Among studies that did not find an improvement in survival for node-positive patients, Duconseil et al.[12] found a lower OS in node-negative patients who received adjuvant chemotherapy. The finding might reflect treatment allocation bias with treatment group harbouring higher rates of adverse prognostic variables including high *T*-stage, node-positive status, and high histologic grade while administered with non-effective chemotherapy [10, 31, 32]. The incongruent results between weighted OS and pooled hazard ratio in the overall comparison may be explained by the use of different studies in each analysis. In turn, this resulted from the use of different survival analysis methods among the included studies.

The role of adjuvant chemotherapy in resectable or borderline resectable PDAC is well established as the standard of care irrespective of nodal status [28, 33–36]. However, the literature for IIPMNs is not so clear. In PDAC, the likely presence of micro-metastatic disease early in the disease course may explain the often, poor prognosis despite *R*0 resection [33] and the improved OS with adjuvant chemotherapy [37]. This may also be true for IIPMNs with metastatic feature, i.e. node positivity. Indeed, stage-matched comparisons of survival outcome between patients with IIPMNs and sporadic PDAC found better survival outcomes for IIPMNs at stage I or IIA (node-negative), while survival outcomes were similar between node-positive and high-grade histology IIPMNs and PDAC [32, 38]. Yet, withholding adjuvant chemotherapy from node-negative IIPMN cannot be recommended based on the meta-analysis findings as the evidence is derived predominantly from retrospective studies. Findings relating to the role of adjuvant chemotherapy in node-negative IIPMNs need to be confirmed by dedicated multicentre prospective studies.

Randomised controlled trials on chemotherapeutic regimens on PDAC had historically focused on gemcitabine-based regimens [35, 36, 39]; however, mFOLFIRINOX (modified fluorouracil and leucovorin, oxaliplatin, and irinotecan) has been widely utilised as first-line therapy following the results of the PRODIGE-24 trial in 2018 [37]. The choice of chemotherapy where reported largely reflected this historic preference for gemcitabine-based regimens. A consequence of the wider use of gemcitabine-based chemotherapy may be an underestimation of efficacy of adjuvant chemotherapy in the treatment of IIPMNs. However, emerging evidence seemed to suggest that there is a limit to generalisability of results from existing trials to other types of pancreatic cancers owing to differences in tumour biology [40, 41]. IIPMNs subtype is a well-established prognostic marker [32, 38, 42]. Among the most common subtypes, tubular carcinoma tends to perform poorly compared to colloid carcinomas [38, 43]. Given the differences in protein expression and genetics, chemotherapy selection and propensity for improved survival may also be dependent on IIPMNs subtype that has yet to be thoroughly explored. In this review, the prevalence of tubular carcinoma ranged widely from 48.9 to 95% and the prevalence of colloid carcinoma ranged from 5 to 66%. The large variance in subtypes prevalence may be a contributor to the high heterogeneity observed in the pooled hazards ratio. The differences in chemotherapeutic regimens used could further explain this heterogeneity too. Future studies must consider interaction between invasive carcinoma subtype (and precursor epithelial type) and specific chemotherapeutic regimes and survival outcomes [44].

The role of radiotherapy as a component of the adjuvant therapy was also not explored but ranged significantly between studies (26.3–100%). Proponents argue that radiotherapy may reduce the risk of local recurrence [29, 45, 46]. Worni et al. published a retrospective study including 972 patients analysing the impact of adjuvant radiotherapy in IIPMN [47]. Adjuvant radiotherapy was associated with improved OS for patients with *T*3/4 tumours and those with node-positive status (HR 0.58, 95% CI 0.41–0.82, *P* = 0.001). However, the study could not identify patients who received chemotherapy in addition to radiotherapy, thereby limiting the utility of its findings [47].

There are several limitations to the present review. The included studies were primarily retrospective in nature with the limitation of retrospective data sets, as reflected in the quality assessment. Low-powered primary outcomes and subgroup outcomes were also a direct result of limitations in study populations. Confounders were present in the adjuvant therapy subgroups and were difficult to address due to the same power limitations even though a quantitative analysis was performed with subgroups in an attempt to limit heterogeneity. The lack of a consistent TNM staging system and differentiation between *R*1 and *R*2 resections in the included studies prevented quantitative analysis within these subgroups. Moreover, paucity of precise information on chemotherapeutic regimen precluded comparison between different types of chemotherapy. Hence, evidence from this review can only suggest adjuvant chemotherapy to be considered but cannot make specific recommendation on the type of chemotherapy to treat IIPMNs. The lack of data on neoadjuvant treatment for IIPMNs meant we could not explore the impact of neoadjuvant therapy for locally advanced IIPMNs. Lastly, diagnosis of IIPMNs was reached without clear exclusion of IPMNs with concomitant PDACs in eight studies [9, 12–16, 25, 26]. Differences in tumour biology and prognosis between the two pathologies likely contributed to heterogeneity in the study population.

## Conclusion

Node-positive patients undergoing pancreatectomy for IIPMNs may have an improved OS with adjuvant chemotherapy. However, marked heterogeneity limited conclusive recommendations for patient selection. Future large multicentre prospective trials are needed to confirm the findings of this study and explore additional predictors of improved OS to guide patient selection.

## Supplementary Information

Below is the link to the electronic supplementary material.

Supplementary file1 (DOCX 16 KB)

## Abbreviations

IPMN: Intraductal papillary mucinous neoplasm
IIPMN: Invasive intraductal papillary mucinous neoplasm
HR: Hazard ratio
OS: Overall survival

## Declarations

### Data availability

The data sets used and/or analysed during the current study are available from the corresponding author on reasonable request.
