# Supplementary material for: Adjuvant Chemotherapy in the Treatment of Intraductal Papillary Mucinous Neoplasms of the Pancreas: Systematic Review and Meta-Analysis
Source: World J Surg. 2021 Sep 20;46(1):223–34. doi: 10.1007/s00268-021-06309-8 (PMC8677688; doi:10.1007/s00268-021-06309-8)
Supplement: Supplementary file 1 — Supplementary file1 (DOCX 16 KB) [file 268_2021_6309_MOESM1_ESM.docx]

**Appendix:**

| **Supplementary table 1**: ROBINS-I Quality Assessment | | | | | | | | |
| --- | --- | --- | --- | --- | --- | --- | --- | --- |
| Study | D1 | D2 | D3 | D4 | D5 | D6 | D7 | Overall |
| Hirono et al. 2020 | Serious | Low | Low | Low | Low | Moderate | Low | Serious |
| Mungo et al. 2020 | Moderate | Low | Low | Low | Moderate | Moderate | Low | Moderate |
| Rodrigues et al. 2020 | Serious | Low | Low | Low | Moderate | Moderate | Low | Serious |
| Marchegiani et al. 2019 | Moderate | Low | Low | Low | Low | Moderate | Low | Moderate |
| Duconseil et al. 2017 | Critical | Low | Low | Low | Low | Moderate | Low | Critical |
| McMillan et al. 2016 | Serious | Low | Low | Low | Low | Moderate | Low | Serious |
| Caponi et al. 2013 | Moderate | Low | Low | Low | Low | Moderate | Low | Moderate |
| Alexander et al. 2011 | Serious | Low | Low | Low | Low | Moderate | Low | Serious |
| Swartz et al. 2010 | Moderate | Low | Low | Low | Low | Moderate | Low | Moderate |
| Turrini et al. 2010 | Moderate | Low | Low | Low | Low | Moderate | Low | Moderate |
| Schnelldorfer et al. 2008 | Critical | Low | Low | Low | Moderate | Moderate | Low | Critical |

D1: Bias due to confounding

D2: Bias due to selection of participants

D3: Bias in classification of interventions

D4: Bias due to deviations from intended interventions

D5: Bias due to missing data

D6: Bias in measurement of outcomes

D7: Bias in selection of the reported result

**Search Strategy**

Intraductal papillary mucinous neoplasm*.mp. OR IPMN.mp OR Pancreatic Intraductal Neoplasm/

AND

(Adjuvant therapy.mp. OR adjuvant therapy.mp. OR Chemotherapy, Adjuvant/ OR chemotherapy.mp. OR Drug Therapy/
